# Meta-analysis of the association between Apolipoprotein E polymorphism and risks of myocardial infarction

**DOI:** 10.1186/s12872-022-02566-0

**Published:** 2022-03-24

**Authors:** Aiyu Shao, Jikang Shi, Zhuoshuai Liang, Lingfeng Pan, Wenfei Zhu, Sainan Liu, Jiayi Xu, Yanbo Guo, Yi Cheng, Yichun Qiao

**Affiliations:** 1 Department of Epidemiology and Biostatistics, School of Public Health, Jilin University, Changchun, 130021 Jilin China; 2 Institute of Translational Medicine, The First Hospital of Jilin University, Changchun, 130021 Jilin China

**Keywords:** Apolipoprotein E polymorphism, Myocardial infarction, Meta-analysis

## Abstract

**Background:**

Myocardial infarction (MI) remains the leading cause of death and disability among cardiovascular diseases worldwide. Studies show that elevated low-density lipid protein cholesterol (LDL-C) levels confer the highest absolute risk of MI, and Apolipoprotein E (ApoE) is implicated in regulating levels of triglycerides (TGs), cholesterol, and LDL-C. Our study aimed to evaluate the association between *APOE* polymorphism and MI, and to provide evidence for the etiology of MI.

**Methods:**

Case–control studies on the association between APOE polymorphisms and the risk of myocardial infarction were included by searching PubMed, Web of Science, and CNKI, and this meta-analysis was written in accordance with PRISMA guideline statement. Odds ratios (ORs) and 95% confidence intervals (CIs) were calculated using either random-effects or fixed-effects models by R software.

**Results:**

A total of 33 eligible articles involving 13,706 cases and 14,817 controls were finally selected. The pooled analysis based on the total eligible articles showed that the risk of MI was associated with *ApoE* epsilon 2 and epsilon 4 alleles. The results showed that patients with MI had a low frequency of the *ε2* allele (OR 0.74, 95% CI 0.64–0.86) and a high frequency of the *ε4* allele (OR 1.24, 95% CI 1.09–1.42).

**Conclusions:**

*APOE ε2*-involved genotypes may be protective factors for MI; in contrast, *ε4*-involved genotypes (*ε4/ε3* vs. *ε3/ε3*, and *ε4/ε4* vs. *ε3/ε3*) may be risk factors for MI.

**Supplementary Information:**

The online version contains supplementary material available at 10.1186/s12872-022-02566-0.

## Introduction

Myocardial infarction (MI) remains the leading cause of death and disability among cardiovascular diseases worldwide [1]. Blood lipid abnormalities are implicated in MI: elevated low-density lipid protein cholesterol (LDL-C) levels confer the highest absolute risk of MI [2]. Apolipoprotein E (ApoE) is implicated in regulating levels of triglycerides (TGs), cholesterol, and LDL-C [3]. Myocardial infarction is usually due to thrombotic occlusion of a coronary vessel caused by the rupture of a vulnerable plaque [4]. Ischemia induces severe ion disturbance in the myocardium [4]. Vulnerable plaques tend to have 30 − 50% stenosis, thin fibrous caps and contain more inflammatory cells such as lipid-laden macrophages [5]. Infiltrated phagocytes clear dead cells and matrix debris, activate anti-inflammatory pathways, and inhibit cytokine and chemokine signaling [4]. Activation of the renin–angiotensin–aldosterone system and release of transforming growth factor-beta promotes the transformation of fibroblasts into myofibroblasts [4].

Epidemiological findings show that the impact of myocardial infarction on global health is significant, with more than one-third of deaths in developed countries [5]. Today, NSTEMI (non-ST-segment elevation myocardial infarction) accounts for 60–75% of all myocardial infarctions. In addition, both in-hospital and 1-year mortality from STEMI (ST-segment elevation myocardial infarction) has declined over the past two decades (5–6% and 7–18%, respectively) [5]. The prevalence of MINOCA (myocardial infarction with no obstructive coronary atherosclerosis) was 6% (95% CI 5–7%), the median age of patients was 55 years (95% CI 51–59 years), and 40% were female. The 12-month mortality in MINOCA patients was 4.7% (95% CI 2.6–6.9%) [6]. The Framingham Heart Study’s 10- year follow-up data revealed that the incidence of MI was 12.9, 38.2, and 71.2 per 1000 in men and 2.2, 5.2, and 13.0 per 1000 in women in the age groups of 30–34, 35–44, and 45–54 years, respectively[7].

The study showed that, regardless of age, more women than men died within one year of the first acute myocardial infarction (AMI) (26% of women and 19% of men respectively) and more women than men died within 5 years of the first AMI (47% of women and 36% of men). At 5 and 10 years after AMI, women had a higher unadjusted mortality rate compared to men and had a 30% readmission rate within 30 days of the first hospitalization, partly due to differences in age, MI risk factors, clinical presentation, and treatment. Women also have a higher prevalence of heart failure and diabetes mellitus (DM) compared to men[8]. A meta-analysis has also shown that myocardial infarction is associated with genotype[9].

The exon 4 of *APOE* has two single nucleotide polymorphisms (SNPs) (rs7412 and rs429358). The two SNPs are used to define the three major alleles of *APOE* (ε2, ε3, and ε4). Allele ε3 possesses cytosines in the amino-acid-coding positions corresponding to rs7412 and rs429358, conferring APOE3 with arginine at residue 158 and cysteine on residue 112 [10]. ε2 arises from substitution rs7412C>T, and rs429358C>T results in ε4. Thus, APOE2 carries cysteine at residue 158 and 112, and APOE4 carries arginine on both positions [11]. Because allele ε3 is the most common in populations, this allele is used as “wild-type”. ε2 and ε4 are used as variants of APOE alleles [12]. The six APOE haplotypes (ε2/ε2, ε2/ε3, ε2/ε4, ε3/ε3, ε3/ε4, and ε4/ε4) are formed by combinations of these three alleles [13].

Associations of *APOE* polymorphism and MI risks have been investigated extensively [14–17]. In 2014, Xu H. et al. performed a meta-analysis, finding that the frequency of MI increases for ε4ε4 *vs.* ε3ε3 (OR 1.59, 95% CI 1.15–2.19, *P* = 0.005); whereas, no significant association exists in ε2ε2 *vs.* ε3ε3 (OR 0.73, 95% CI 0.40–1.32, *P* = 0.29) [18]. In contrast, a meta-analysis issued in 2015 revealed that, for ε2ε2 *vs.* ε3ε3, a decreased frequency of MI exists (OR 0.40, 95% CI 0.20–0.83, *P* = 0.00), except in Caucasian and Asian populations, and no significant association exists in ε4ε4 *vs.* ε3ε3 (OR 1.34, 95% CI 0.91–1.98, *P* = 0.186) in these populations [19].

Possible reasons for the above results are: (1) they had different inclusion and exclusion criteria: Xu H. et al.'s study in 2014 did not consider cancer risk, but such studies were included in the 2015 article, further led to a large difference in the number of articles finally included in the study between the two: in 2014 (n = 33); in 2015 (n = 22); (2) the results of 2015 divided the ethnic group into three subgroups and found that Caucasians and Asians have different gene expression frequencies compared to other ethnic groups. But 2014 results only compared two subgroups of Caucasians and Asians. Thus, we conducted an up-to-date meta-analysis to resolve these conflicting results.

## Materials and methods

### Search strategy

According to the PRISMA guideline, we searched all articles published before May 1, 2021, from both English databases (PubMed, and Web of Science database) and Chinese databases (CNKI database) using the combination of keywords (“Apolipoprotein E” OR “ApoE” OR “APOE” AND “myocardial infarction” OR “MI” AND “polymorphism” OR “polymorphisms” OR “variants” OR “variant”). In addition, we searched related articles that had not been included in the initial search using Google (www.google.com).

### Inclusion and exclusion criteria

Articles were included for further selection if they fulfilled the inclusion criteria: (1) articles issued in English or Chinese were performed under either hospital-based or population-based design; (2) evaluation of the association between *APOE* polymorphisms and MI was involved and the data can be extracted in articles; and (3) odds ratios (ORs) with 95% confidence intervals (CIs) were evaluated or sufficient data were suggested to assess associations. Articles were removed according to the exclusion criteria: (1) non-English or non-Chinese articles; (2) abstracts, conference records, systematic reviews or meta-analysis, and articles without case–control studies; (3) articles with insufficient data to calculate the ORs and 95% CIs; (4) the data originated from the online dataset; (5) articles lacking usable data on genotypes or allele frequencies; and “star”, which was delimited in the 2.3 section judged (6) low-quality articles.

### Data extraction and quality assessment

All included articles were identified by two investigators (Jikang Shi and Zhuoshuai Liang). If the two investigators could not agree on an included article, the third investigator (Lingfeng Pan) settled in conformity finally. We collected the following data (first author's name, publication year, ethnicity, distribution of genotypes and alleles in MI cases and controls, sample sizes of MI cases and controls, and evidence of conforming to the Hardy–Weinberg equilibrium (HWE) among controls). The other information was extracted, such as sex and the last name of the first author. We evaluated the quality of the included articles using the Newcastle–Ottawa scale (NOS). It allocated a score of one point when an included article met a condition; otherwise, no point (0 scores) was allocated. Furthermore, for each included article, the sum of all points (total Quality Score) represented the quality of this article [20]. Low-quality articles were also excluded to avoid selection bias.

### Statistical analysis

The association of *APOE* polymorphisms and myocardial infarction was analyzed using R Studio (Version 1.1.383) (RStudio, Inc., MA, USA). We designated the ε3 allele and ε3/ε3 as the reference and collected the ORs and 95% CIs for evaluating the prognostic value of *APOE* polymorphisms. The pooled ORs and 95%CIs were estimated in the seven types (*ε2/ε2 vs. ε3/ε3*, *ε2/ε3 vs. ε3/ε3*, *ε2/ε4 vs. ε3/ε3*, *ε4ε3 vs. ε3/ε3*, *ε4/ε4 vs. ε3/ε3, ε2* allele *vs. ε3* allele, and *ε4* allele *vs. ε3* allele).

Hardy–Weinberg equilibrium (HWE) for each included article among control groups was evaluated using the Chi-square test of goodness, and HWE was rejected if *P* < 0.05. ORs and 95% CIs were used to assess the strength of association between *APOE* polymorphisms and MI risks. Heterogeneity sources were investigated based on the HWE test (Yes or No), score (< 6 or ≥ 6), and subgroup analysis for ethnicity (Asian or Other). Both Chi-square test-based Q-statistic and *I*^2^-statistic were utilized to evaluate heterogeneity. We carried out the comparisons of *APOE* genotypes, as genotypes can represent the combined effect of alleles. For heterogeneity between studies given by *I*^2^ > 50%, random-effect models were applied; otherwise, if *I*^2^ < 50%, fixed-effect models were used [21]. Furthermore, sensitivity analysis was used to assess the stability of articles. The publication bias of this meta-analysis was analyzed using funnel plot and Begg's test [22].

### Trial sequential analysis (TSA)

Traditional meta-analysis is criticized because the data of articles are inevitably clinically diverse among patients, such as ethnicities and diseases states. Systematic bias and random errors result in false-positive results (type I errors) or overestimated treatment effects that may also be obtained by Meta-analyses. Because of neglecting heterogeneity, simply pooling the results is inappropriate [23].

Trial sequential analysis (TSA) provides the required sample size (RIS), analyzing monitoring boundaries of trial sequential if articles do not reach the RIS [24]. The horizontal ordinate is the sample size, and the vertical ordinate is the Z-curve score of the effect. The Z-curve in the upper half of the vertical ordinate indicates a protective effect. Rather, that in the lower half of the vertical ordinate indicates risk effect. The fewer participants and events are, the more restrictive the monitoring boundaries are needed. Furthermore, a much less *P*-value is required to obtain statistical significance [22]. TSA software (TSA, version 0.9.5.5; Copenhagen Trial Unit, Copenhagen, Denmark, 2016) was used in this Meta-analysis. We set type I error as 5% and type II error as 20% [23]; thus, the statistical power was 80% (power = 1–20%). The relative risk reduction (RRR) was defined as 20%.

## Results

### Characteristics of studies

**Fig. 1:**
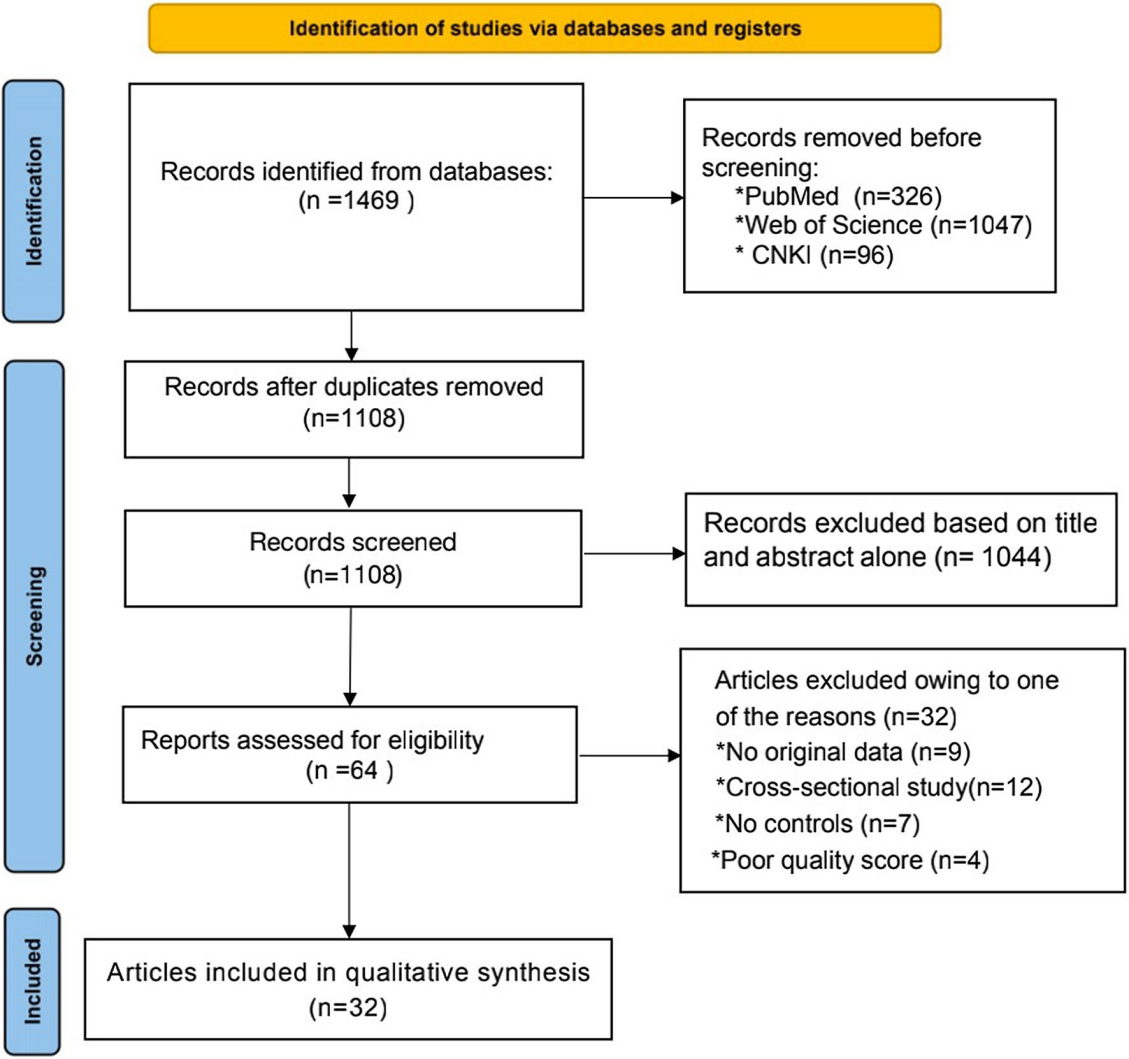
Flow chart of the process for literature identification and selection

We scrutinized 1469 articles according to the inclusion and exclusion criteria, finally selecting 32 articles investigated in this meta-analysis [16, 25–51]. The selected 32 articles provided 13,706 cases with MI and 14,817 controls. (Fig. 1; Table 1).

**Table 1:** Main characteristics of the included studies

Study	Year	Country	Ethnicity	Sample size		Quality	HWE	ApoE ε2 (n)		ApoE ε3 (n)		ApoE ε4 (n)	
				Case	Control	Score	Y/N	Case	Control	Case	Control	Case	Control
Cumming et al	1984	Scotland	Scottish	239	239	7	Y (*P* = 0.57)	28	39	351	367	99	70
Yamamura et al	1984	Germany	Caucasian	523	1031	6	N (*P* < 0.01)	93	379	826	1594	127	09
Utermann et al	1984	Japan	Japanese	523	1031	5	N (*P* = 0.01)	93	379	826	1594	127	309
Lenzen et al	1986	Germany	Caucasian	570	624	8	Y (*P* = 0.16)	63	99	907	978	170	171
Luc et al	1994	Belfast	Caucasian	183	176	7	Y (*P* = 0.57)	25	36	270	266	71	50
Luc et al	1994	Lille	Caucasian	64	150	7	Y (*P* = 0.98)	6	33	105	223	17	44
Luc et al	1994	Strasbourg	Caucasian	187	172	7	Y (*P* = 0.51)	27	29	288	274	59	41
Luc et al	1994	Toulouse	Caucasian	140	182	7	Y (*P* = 0.84)	16	20	228	311	36	33
Joven et al	1998	Spain	Caucasian	250	250	6	Y (*P* = 0.19)	39	25	397	438	64	37
Nakai et al	1998	Japan	Japanese	254	422	6	Y (*P* = 0.29)	12	20	418	744	66	80
Batalla et al	2000	Spain	Spainish	220	200	8	Y (*P* = 0.89)	10	19	389	348	41	33
Zhao et al	2000	Liaoning	Asian	50	49	7	Y (*P* = 0.76)	4	5	90	90	6	3
Raslová et al	2001	Slovak	Caucasian	71	71	6	Y (*P* = 0.30)	12	7	111	114	13	17
Wang et al	2001	Xinjiang	Asian	54	106	6	Y (*P* = 0.58)	3	15	82	174	23	23
Gong et al	2001	Guangdong	Asian	108	115	7	Y (*P* = 0.47)	14	16	170	196	32	18
Bai et al	2001	Liaoning	Asian	47	113	6	Y (*P* = 0.36)	4	11	90	200	6	9
Kolovou et al	2002	Greece,	Greek	267	240	7	Y (*P* = 0.72)	39	39	412	392	83	49
Mamotte et al	2002	Australia	Caucasian	359	639	6	Y (*P* = 1.54)	39	92	554	983	125	203
Kumar et al	2003	North India	Indian	35	45	5	N (*P* = 0.03)	7	13	36	73	27	4
Li et al	2003	Nantong	Asian	67	152	5	Y (*P* = 0.10)	16	26	98	253	22	25
Chen et al	2003	Liaoning	Asian	50	110	5	Y (*P* = 0.09)	4	11	90	92	6	3
Keavney et al	2004	UK	Caucasian	4484	5757	6	N (*P* < 0.01)	440	686	6778	8830	1206	1376
Ranjith et al	2004	Indian	African	195	300	6	N (*P* < 0.01)	10	27	330	517	50	56
Aasvee et al	2006	estonia	Caucasian	71	85	8	Y (*P* = 0.98)	7	18	110	133	23	21
Baum et al	2006	Hongkong	chinese	231	311	6	Y (*P* = 0.81)	17	70	387	505	58	47
Koch et al	2008	Germany	Caucasian	3657	1211	6	Y (*P* = 0.72)	517	201	5769	1899	1028	322
Viitanen et al	2011	Finland	Caucasian	118	110	5	Y (*P* = 0.98)	7	10	171	175	58	35
Onrat et al	2012	Turkey	Turkish	100	36	6	Y (*P* = 0.55)	12	4	172	62	16	6
Tanguturi et al	2013	India	Indian	202	210	8	Y (*P* = 0.18)	12	17	329	371	63	32
Kukava et al	2017	Russia	Russians	405	198	7	Y (*P* = 0.50)	68	32	698	326	44	38
Gupta et al	2018	India	Indian	168	89	6	Y (*P* = 0.54)	18	4	302	165	16	9
Hu et al	2020	Jiangxi	Asian	53	632	7	N (*P* = 0.02)	128	28	1055	83	81	23

### Quantitative synthesis

**Fig. 2:**
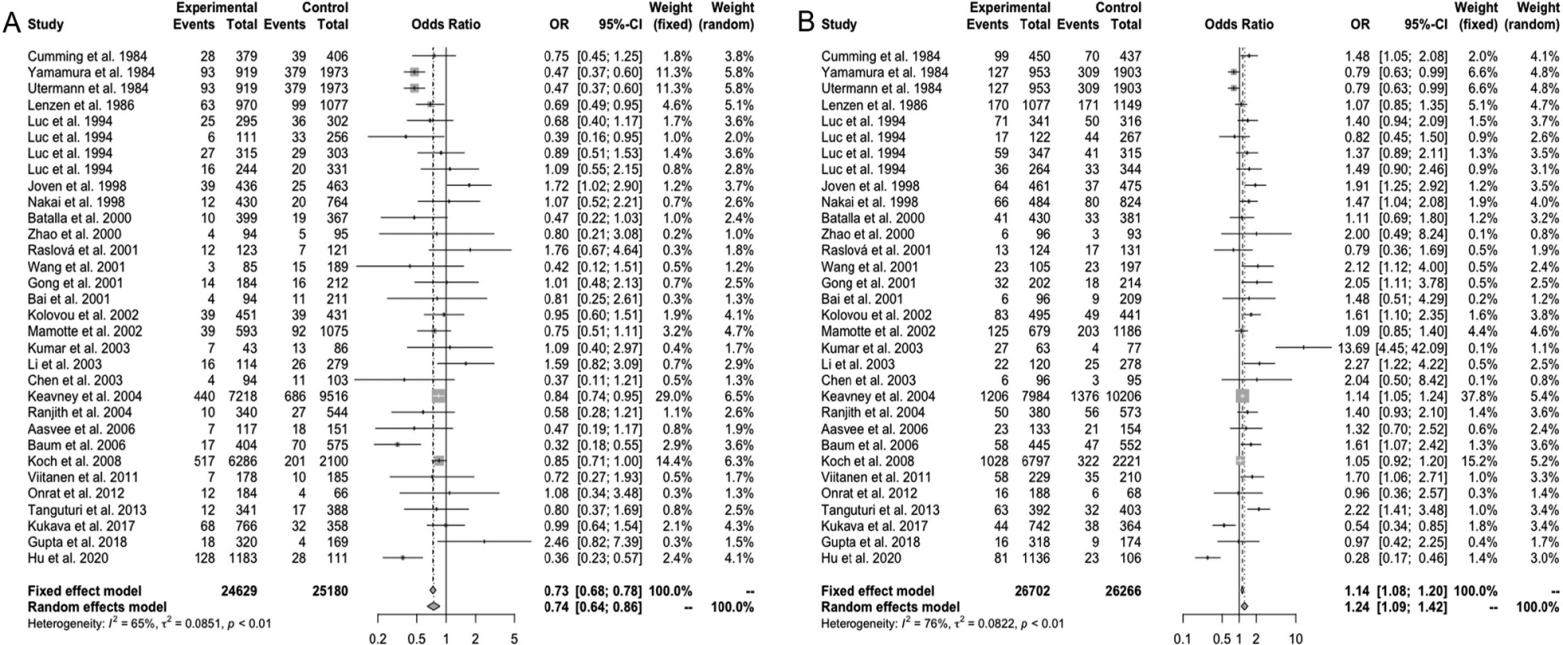
Forest plot for the association between myocardial infarction risk and *APOE* ε2 allele vs. ε3 allele (**A**); forest plot for the association between myocardial infarction risk and *APOE* ε4 allele vs. ε3 allele (**B**)

**Fig. 3:**
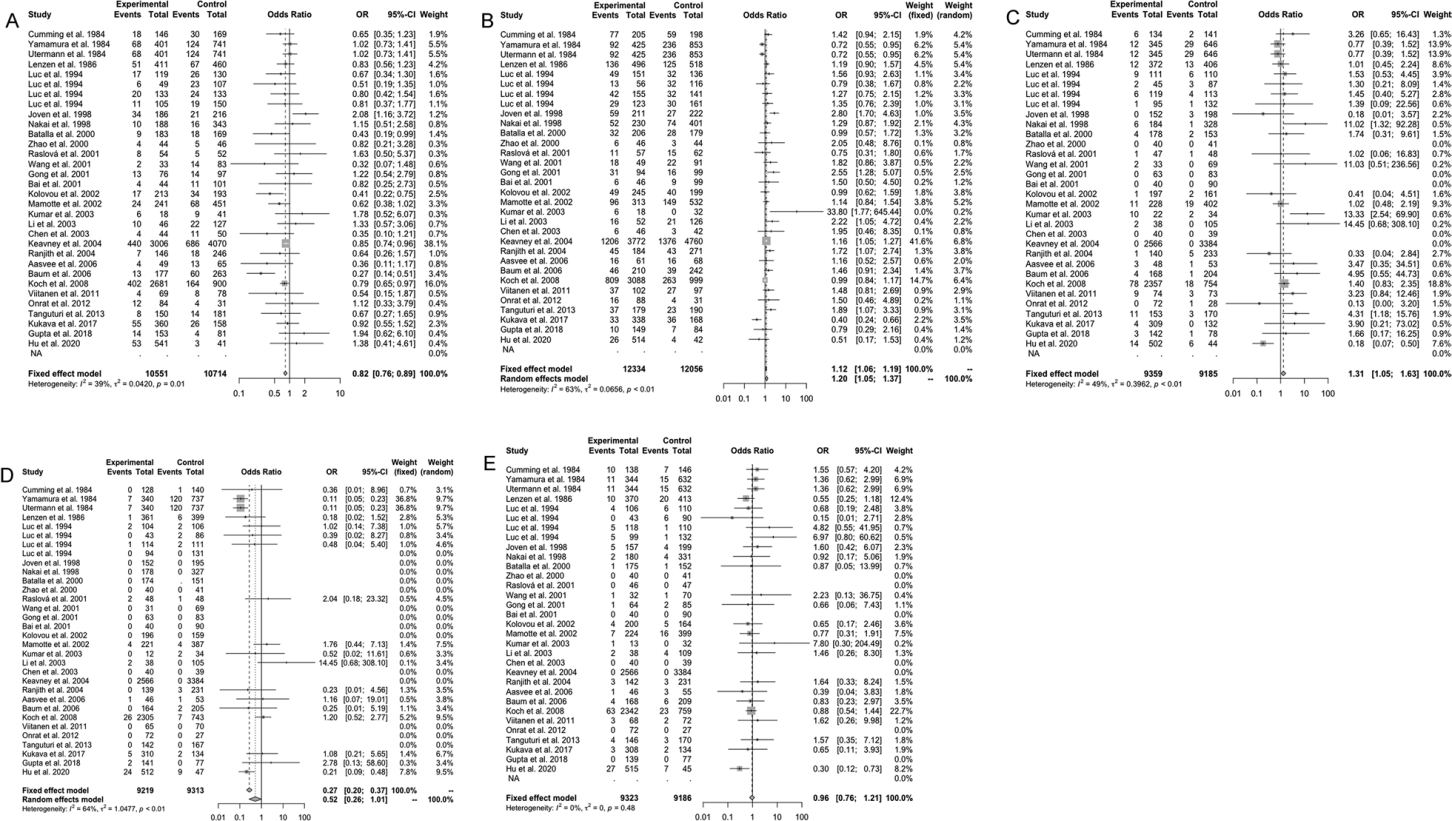
Forest plot for association between *APOE* polymorphism and MI risks in genotypes: **A** *ε2/ε3* vs. *ε3/ε3*; **B** *ε3/ε4* vs. *ε3/ε3*; **C** *ε4/ε4* vs. *ε3/ε3*; **D** *ε2/ε2* vs. *ε3/ε3*; **E** *ε2/ε4* vs. *ε3/ε3*

In the pooled analysis, the significant heterogeneity between *APOE* polymorphism and MI risks was found in *ε2 vs. ε3* (*I*^2^ = 65%, *P* < 0.01) and *ε4 vs. ε3* (*I*^2^ = 76%, *P* < 0.01). The random-effects model revealed that patients with MI had a low frequency of the *ε2* (OR 0.74, 95% CI 0.64–0.86, *P* < 0.01) (Fig. 2A) and a high frequency of the *ε4* (OR 1.24, 95% CI 1.09–1.42, *P* < 0.01) (Fig. 2B); the pooled OR of *ε2/ε3* vs. *ε3/ε3* was 0.82 (95% CI 0.76–0.89, *P* = 0.01) (Fig. 3A); the pooled OR of *ε3/ε4* vs. *ε3/ε3* was OR 1.20 (95% CI 1.05–1.37, *P* < 0.01) (Fig. 3B); and the pooled OR of *ε4/ε4* vs. *ε3/ε3* was OR = 1.31 (95% CI 1.05–1.63, *P* < 0.01) (Fig. 3C). However, compared with *ε3/ε3*, *ε2/ε2* (Fig. 3D) and *ε2/ε4* (Fig. 3E) might not influence MI risks (for *ε2/ε2*, OR 0.52, 95% CI 0.26–1.01, *P* < 0.01) (for *ε2/ε4*, OR 0.96, 95% CI 0.76–1.21, *P* = 0.48).

### Subgroup analysis

To find the potential source of heterogeneity, we ran meta-regression analysis before subgroup analysis, The results show that HWE is a source of heterogeneity in ε4 vs. ε3(P = 0.019); in ε3/ε4 vs. ε3/ε3, both HWE (*P* = 0.0025)and ethnicity (*P* = 0.0294)are sources of heterogeneity.

We performed subgroup analysis based on the HWE, finding that articles satisfying the HWE had significant heterogeneity. Furthermore, we found that low MI risks existed in carriers of the *ε2* allele (OR 0.82, 95% CI 0.74–0.90, *P* = 0.01) and those of *ε2/ε3* vs. *ε3/ε3* (OR 0.75, 95% CI 0.67–0.85, *P* < 0.01); in contrast, high MI risks existed in carriers of the *ε4* allele (OR 1.34, 95% CI 1.18–1.52, *P* < 0.01) and those of *ε3/ε4* vs. *ε3/ε3* (OR 1.27, 95% CI 1.09–1.48, *P* < 0.01). In addition, articles not satisfying the HWE had significant heterogeneity (for *ε2* allele, *P* < 0.01; for *ε4* allele, *P* < 0.01; for *ε2/ε4* vs. *ε3/ε3*, *P* = 0.04; for *ε3/ε4* vs. *ε3/ε3*, *P* < 0.01; and for *ε4/ε4* vs. *ε3/ε3*, *P* < 0.01). Moreover, we found that low MI risks existed in carriers of the *ε2* allele (OR 0.56, 95% CI 0.40–0.79, *P* < 0.01), but there were no associations of MI risks with carriers of *ε4* allele or with those of *ε4*-involved (*ε2/ε4* vs. *ε3/ε3,* and *ε3/ε4* vs. *ε3/ε3*) genotypes.

We carried out subgroup analysis based on ethnicity, finding that articles involving Asians had significant heterogeneity. The *ε2* allele was a protective factor for MI (*P* < 0.01, OR 0.70, 95% CI 0.50–0.98); in contrast, the *ε4* allele (*P* < 0.01, OR 1.56, 95% CI 1.04–2.35) and *ε4/ε3* vs. *ε3/ε3* (*P* < 0.01, OR 1.44, 95% CI 1.03–2.01) were risk factors for MI. In addition, there were no significant associations of MI risks with carriers of *ε2/ε4* vs. *ε3/ε3* (*P* = 0.27), with those of *ε2/ε2* vs. *ε3/ε3* (OR 0.38, 95% CI 0.12–1.20, *P* = 0.16), with those of *ε2/ε3* vs. *ε3/ε3* (OR 0.85, 95% CI 0.68–1.03, *P* = 0.34), or with those of *ε4/ε4* vs. *ε3/ε3* (OR 2.90, 95% CI 0.91–9.23, *P* = 0.48). Furthermore, we found that articles involving other ethnicities had significant heterogeneity. The *ε2* allele was a protective factor for MI (*P* < 0.01, OR 0.78, 95% CI 0.67–0.91); on the contrary, the *ε4* allele was a risk factor for MI (*P* < 0.01, OR 1.16, 95% CI 1.04–1.30). There was no significant heterogeneity of MI risks with carriers of *ε2/ε3* vs. *ε3/ε3* (*P* = 0.09), with those of *ε2/ε4* vs. *ε3/ε3* (*P* = 0.55), or with those of *ε4/ε4* vs. *ε3/ε3* (*P* = 0.71). There was no significant association of MI risks with carriers of *ε2/ε2* vs. *ε3/ε3* (OR 0.59, 95% CI 0.26–1.36, *P* = 0.09) or with those of *ε3/ε4* vs. *ε3/ε3* (OR 1.13, 95% CI 0.97–1.31, *P* = 0.63).

We carried out subgroup analysis based on the score, finding that articles satisfying the high score had no heterogeneity of MI risks with carriers of the *ε2* allele (*P* > 0.05) or with those of *ε2*-involved genotypes (all *P* > 0.05). There was no significant association of MI risks with carriers of *ε4* vs. *ε3* (*P* < 0.01, OR 1.17, 95% CI 0.90–1.53), with those of *ε3/ε4* vs. *ε3/ε3* (*P* < 0*.*01, OR 1.16, 95% CI 0.91–1.47), or with those of *ε4/ε4* vs. *ε3/ε3* (*P* = 0*.*03, OR 1.32, 95% CI 0.89–1.94). In addition, articles not satisfying the low score showed that all genotypes had significant heterogeneity (all *P* < 0.01). Low MI risks existed in carriers of the *ε2* allele (*P* < 0.01, OR 0.78, 95% CI 0.63–0.97); in contrast, high MI risks existed in carriers of the *ε4* allele (*P* < 0.01, OR 0.78, 95% CI 1.09–1.50) or in those of *ε3/ε4* vs. *ε3/ε3* (*P* < 0.01, OR 1.22, 95% CI 1.03–1.45). There were no significant associations of MI risks with carriers of *ε2/ε2* vs. *ε3/ε3* (OR 1.22, 95% CI 1.03–1.4, *P* > 0.05), with those of *ε2/ε3* vs. *ε3/ε3* (OR 0.87, 95% CI 0.72–1.60, *P* > 0.05), or with those of *ε4/ε4* vs. *ε3/ε3* (OR 1.53, 95% CI 0.91–2.59) (Table 2).

**Table 2:** Subgroup analysis of associations of MI risks with *APOE* alleles or with genotypes

Variable	Asian		Other	
	*OR* (95% CI)	*I*^2^ (%)	*OR* (95%CI)	*I*^2^ (%)
Alleles				
ε2	0.70 (0.50,0.98)	66	0.78 (0.67,0.91)	55
ε4	1.56 (1.04,2.35)	86	1.16 (1.04,1.30)	57
Genotypes				
ε2/ε2	0.38 (0.12,1.20)	62	0.59 (0.26, 1.36)	61
ε2/ε3	0.85 (0.60, 1.22)	50	0.82 (0.75, 0.90)	32
ε2/ε4	0.96 (0.61, 1.51)	19	0.96 (0.74, 1.25)	0
ε3/ε4	1.44 (1.03, 2.01)	64	1.13 (0.97, 1.31)	64
ε4/ε4	2.90 (0.91, 9.23)	79	1.19 (0.92, 1.55)	0

ε2/ε2, ε2/ε3, ε2/ε4, ε3/ε4 and ε4/ε4 were compared with ε3/ε3. ε2 and ε4 were compared with ε3

### Sensitivity analysis

**Fig. 4:**
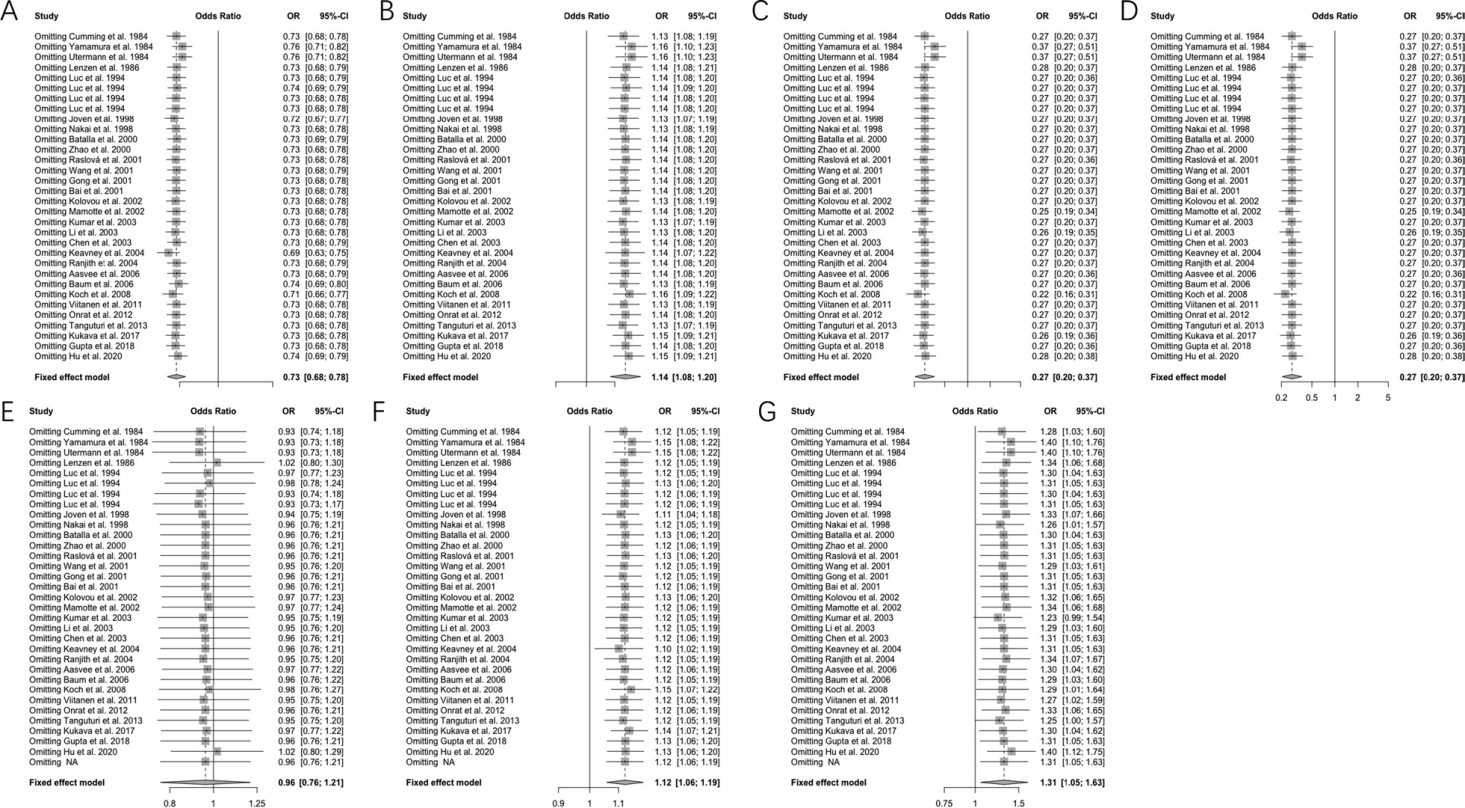
Forest plot of subgroup analysis of the association between *APOE* alleles/genotypes and myocardial infarction

To clarify the sources of heterogeneity, sensitivity analyses were performed to assess the stability of the results and the source of the heterogeneity by omitting individual studies and to show the influence of the individual data on the total ORs. Results of sensitivity analysis on the *ε2* allele (Fig. 4A), the *ε4* allele (Fig. 4B), *ε2/ε2* vs. *ε3/ε3* (Fig. 4C), *ε2/ε3* vs. *ε3/ε3* (Fig. 4D), *ε2/ε4* vs. *ε3/ε3* (Fig. 4E), *ε3/ε4* vs. *ε3/ε3* (Fig. 4F), and *ε4/ε4* vs. *ε3/ε3* (Fig. 4G) were presented in Fig. 4. No individual article affected the corresponding pooled ORs and 95%CIs; therefore, the result of this meta-analysis was statistically robust (Tables 3, 4).

**Table 3:** Sensitivity analysis of associations between *APOE* alleles and MI risks

Study	ε2	ε4
Cumming et al	0.73 (0.68, 0.78)	1.13 (1.08, 1.19)
Yamamura et al	0.76 (0.71, 0.82)	1.16 (1.10, 1.23)
Utermann et al	0.76 (0.71, 0.82)	1.16 (1.10, 1.23)
Lenzen et al	0.73 (0.68, 0.79)	1.14 (1.08, 1.21)
Luc et al	0.73 (0.68, 0.79)	1.14 (1.08, 1.20)
Luc et al	0.74 (0.69, 0.79)	1.14 (1.09, 1.20)
Luc et al	0.73 (0.68, 0.78)	1.14 (1.08, 1.20)
Luc et al	0.73 (0.68, 0.78)	1.14 (1.08, 1.20)
Joven et al	0.72 (0.67, 0.77)	1.13 (1.07, 1.19)
Nakai et al	0.73 (0.68, 0.78)	1.13 (1.08, 1.19)
Batalla et al	0.73 (0.69, 0.79)	1.14 (1.08, 1.20)
Zhao et al	0.73 (0.68, 0.78)	1.14 (1.08, 1.20)
Raslová et al	0.73 (0.68, 0.78)	1.14 (1.08, 1.20)
Wang et al	0.73 (0.68, 0.79)	1.14 (1.08, 1.20)
Gong et al	0.73 (0.68, 0.78)	1.14 (1.08, 1.20)
Bai et al	0.73 (0.68, 0.78)	1.14 (1.08, 1.20)
Kolovou et al	0.73 (0.68, 0.78)	1.13 (1.08, 1.19)
Mamotte et al	0.73 (0.68, 0.78)	1.14 (1.08, 1.20)
Kumar et al	0.73 (0.68, 0.78)	1.13 (1.07, 1.19)
Li et al	0.73 (0.68, 0.78)	1.13 (1.08, 1.20)
Chen et al	0.73 (0.68, 0.79)	1.14 (1.08, 1.20)
Keavney et al	0.69 (0.63, 0.75)	1.14 (1.07, 1.22)
Ranjith et al	0.73 (0.68, 0.79)	1.14 (1.08, 1.20)
Aasvee et al	0.73 (0.68, 0.79)	1.14 (1.08, 1.20)
Baum et al	0.74 (0.69, 0.80)	1.13 (1.08, 1.19)
Koch et al	0.71 (0.66, 0.77)	1.16 (1.09, 1.22)
Viitanen et al	0.73 (0.68, 0.78)	1.13 (1.08, 1.19)
Onrat et al	0.73 (0.68, 0.78)	1.14 (1.08, 1.20)
Tanguturi et al	0.73 (0.68, 0.78)	1.13 (1.07, 1.19)
Kukava et al	0.73 (0.68, 0.78)	1.15 (1.09, 1.21)
Gupta et al	0.73 (0.68, 0.78)	1.14 (1.08, 1.20)
Hu et al	0.74 (0.69, 0.79)	1.15 (1.09, 1.21)

ε2 and ε4 were compared with ε3

**Table 4:** Sensitivity analysis of associations between *APOE* genotypes and MI risks

Study	ε2/ε2	ε2/ε3	ε2/ε4	ε3/ε4	ε4/ε4
Cumming et al	0.27 (0.20, 0.37)	0.27 (0.20, 0.37)	0.93 (0.74, 1.18)	1.12 (1.05, 1.19)	1.28 (1.03, 1.60)
Yamamura et al	0.37 (0.27, 0.51)	0.37 (0.27, 0.51)	0.93 (0.73, 1.18)	1.15 (1.08, 1.22)	1.40 (1.10, 1.76)
Utermann et al	0.37 (0.27, 0.51)	0.37 (0.27, 0.51)	0.93 (0.73, 1.18)	1.15 (1.08, 1.22)	1.40 (1.10, 1.76)
Lenzen et al	0.28 (0.20, 0.37)	0.28 (0.20, 0.37)	1.02 (0.80, 1.30)	1.12 (1.05, 1.19)	1.34 (1.06, 1.68)
Luc et al	0.27 (0.20, 0.36)	0.27 (0.20, 0.36)	0.97 (0.77, 1.23)	1.12 (1.05, 1.19)	1.30 (1.04, 1.63)
Luc et al	0.27 (0.20, 0.37)	0.27 (0.20, 0.37)	0.98 (0.78, 1.24)	1.13 (1.06, 1.20)	1.31 (1.05, 1.63)
Luc et al	0.27 (0.20, 0.37)	0.27 (0.20, 0.37)	0.93 (0.74, 1.18)	1.12 (1.06, 1.19)	1.30 (1.04, 1.63)
Luc et al	0.27 (0.20, 0.37)	0.27 (0.20, 0.37)	0.93 (0.73, 1.17)	1.12 (1.06, 1.19)	1.31 (1.05, 1.63)
Joven et al	0.27 (0.20, 0.37)	0.27 (0.20, 0.37)	0.94 (0.75, 1.19)	1.11 (1.04, 1.18)	1.33 (1.07, 1.66)
Nakai et al	0.27 (0.20, 0.37)	0.27 (0.20, 0.37)	0.96 (0.76, 1.21)	1.12 (1.05, 1.19)	1.26 (1.01, 1.57)
Batalla et al	0.27 (0.20, 0.37)	0.27 (0.20, 0.37)	0.96 (0.76, 1.21)	1.13 (1.06, 1.20)	1.30 (1.04, 1.63)
Zhao et al	0.27 (0.20, 0.37)	0.27 (0.20, 0.37)	0.96 (0.76, 1.21)	1.12 (1.06, 1.19)	1.31 (1.05, 1.63)
Raslová et al	0.27 (0.20, 0.36)	0.27 (0.20, 0.36)	0.96 (0.76, 1.21)	1.13 (1.06, 1.20)	1.31 (1.05, 1.63)
Wang et al	0.27 (0.20, 0.37)	0.27 (0.20, 0.37)	0.95 (0.76, 1.20)	1.12 (1.05, 1.19)	1.29 (1.03, 1.61)
Gong et al	0.27 (0.20, 0.37)	0.27 (0.20, 0.37)	0.96 (0.76, 1.21)	1.12 (1.05, 1.19)	1.31 (1.05, 1.63)
Bai et al	0.27 (0.20, 0.37)	0.27 (0.20, 0.37)	0.96 (0.76, 1.21)	1.12 (1.06, 1.19)	1.31 (1.05, 1.63)
Kolovou et al	0.27 (0.20, 0.37)	0.27 (0.20, 0.37)	0.97 (0.77, 1.23)	1.13 (1.06, 1.20)	1.32 (1.06, 1.65)
Mamotte et al	0.25 (0.19, 0.34)	0.25 (0.19, 0.34)	0.97 (0.77, 1.24)	1.12 (1.06, 1.19)	1.34 (1.06, 1.68)
Kumar et al	0.27 (0.20, 0.37)	0.27 (0.20, 0.37)	0.95 (0.75, 1.19)	1.12 (1.05, 1.19)	1.23 (0.99, 1.54)
Li et al	0.26 (0.19, 0.35)	0.26 (0.19, 0.35)	0.95 (0.76, 1.20)	1.12 (1.05, 1.19)	1.29 (1.03, 1.60)
Chen et al	0.27 (0.20, 0.37)	0.27 (0.20, 0.37)	0.96 (0.76, 1.21)	1.12 (1.06, 1.19)	1.31 (1.05, 1.63)
Keavney et al	0.27 (0.20, 0.37)	0.27 (0.20, 0.37)	0.96 (0.76, 1.21)	1.10 (1.02, 1.19)	1.31 (1.05, 1.63)
Ranjith et al	0.27 (0.20, 0.37)	0.27 (0.20, 0.37)	0.95 (0.75, 1.20)	1.12 (1.05, 1.19)	1.34 (1.07, 1.67)
Aasvee et al	0.27 (0.20, 0.36)	0.27 (0.20, 0.36)	0.97 (0.77, 1.22)	1.12 (1.06, 1.19)	1.30 (1.04, 1.62)
Baum et al	0.27 (0.20, 0.37)	0.27 (0.20, 0.37)	0.96 (0.76, 1.22)	1.12 (1.05, 1.19)	1.29 (1.03, 1.60)
Koch et al	0.22 (0.16, 0.31)	0.22 (0.16, 0.31)	0.98 (0.76, 1.27)	1.15 (1.07, 1.22)	1.29 (1.01, 1.64)
Viitanen et al	0.27 (0.20, 0.37)	0.27 (0.20, 0.37)	0.95 (0.75, 1.20)	1.12 (1.05, 1.19)	1.27 (1.02, 1.59)
Onrat et al	0.27 (0.20, 0.37)	0.27 (0.20, 0.37)	0.96 (0.76, 1.21)	1.12 (1.06, 1.19)	1.33 (1.06, 1.65)
Tanguturi et al	0.27 (0.20, 0.37)	0.27 (0.20, 0.37)	0.95 (0.75, 1.20)	1.12 (1.05, 1.19)	1.25 (1.00, 1.57)
Kukava et al	0.26 (0.19, 0.36)	0.26 (0.19, 0.36)	0.97 (0.77, 1.22)	1.14 (1.07, 1.21)	1.30 (1.04, 1.62)
Gupta et al	0.27 (0.20, 0.36)	0.27 (0.20, 0.36)	0.96 (0.76, 1.21)	1.13 (1.06, 1.20)	1.31 (1.05, 1.63)
Hu et al	0.28 (0.20, 0.38)	0.28 (0.20, 0.38)	1.02 (0.80, 1.29)	1.13 (1.06, 1.20)	1.40 (1.12, 1.75)

ε2/ε2, ε2/ε3, ε2/ε4, ε3/ε4 and ε4/ε4 were compared with ε3/ε3

### Publication bias

Funnel plots were performed to assess the publication bias and quantified by Begg’s test. The results showed that there was no significant publication bias in neither alleles nor genotypes (all *P* > 0.05) (Additional file 1: Figure S1).

### TSA

For associations of MI risks with *ε2* allele (Additional file 2: Figure S2A), with *ε2/ε2* vs. *ε3/ε3* (Additional file 2: Figure S2B), and with *ε2/ε3* vs. *ε3/ε3* (Additional file 2: Figure S2C), simple sizes reached RIS, and Z-curves crossed the trial sequential monitoring boundaries. For associations of MI risks with *ε4* allele (Additional file 3: Figure S3A), with *ε3/ε4* vs. *ε3/ε3* (Additional file 3: Figure S3B), and with *ε4/ε4* vs. *ε3/ε3* (Additional file 3: Figure S3C), simple sizes reached the RIS but Z-curves did not crosse the trial sequential monitoring boundaries. For associations of MI risks with *ε2/ε4* vs. *ε3/ε3*, simple size neither reached the RIS nor Z-curves crosse the trial sequential monitoring boundaries (Additional file 4: Figure S4). Thus, the *ε2* allele and *ε2*-involved genotypes were protective factors for MI; in contrast, the *ε4* allele and *ε4*-involved genotypes (*ε4/ε3* vs. *ε3/ε3*, and *ε4/ε4* vs. *ε3/ε3*) were risk factors for MI. There was no significant association between MI risks and genotype *ε2/ε4*.

## Discussion

This meta-analysis, based on up-to-date data, further investigate the association between APOE polymorphism and MI risks, indicating that the *ε2* allele and *ε2*-involved genotypes may be protective factors for MI; in contrast, the *ε4* allele and *ε4*-involved genotypes (*ε4/ε3* vs. *ε3/ε3*, and *ε4/ε4* vs. *ε3/ε3*) may be risk factors for MI.

**Fig. 5:**
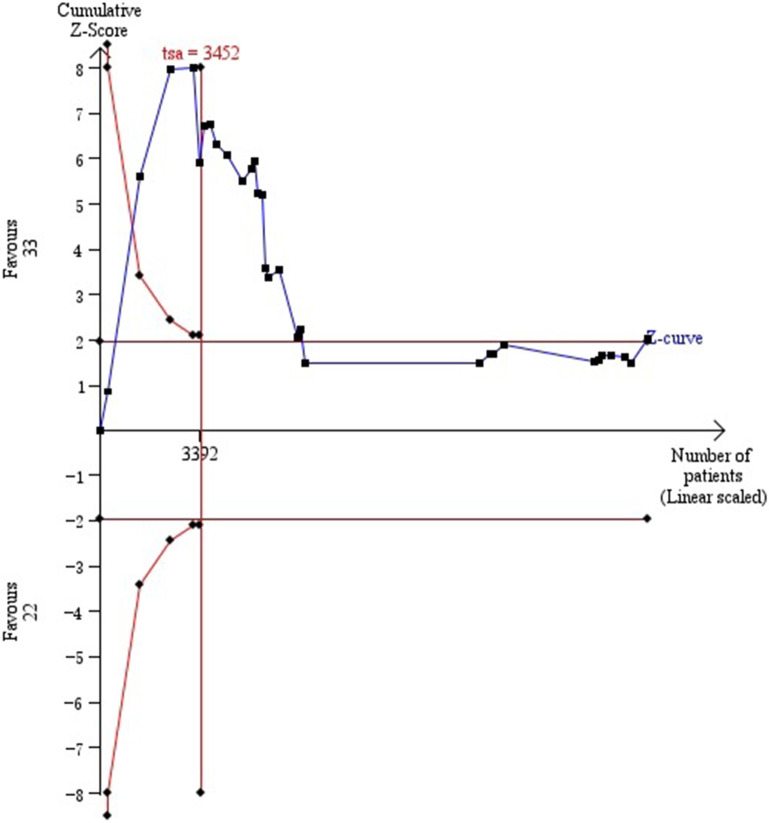
Trial sequential analysis of the association between *ApoE* genotype ε2/ε2 vs. ε3/ε3 and myocardial infarction

We found that the genotype *ε2/ε2* is associated with MI risks. Of note, Qi et al. observed the genotype *ε2/ε2* is not associated with MI risks [53]. Apart from methods that Qi et al. used [53], we adopted TSA additionally. Simple sizes reached RIS, and Z-curves crossed the trial sequential monitoring boundaries, documenting that the association of the genotype *ε2/ε2* with MI risks is robust (Fig. 5).

Both the meta-analysis of Luc [29] and our meta-analysis identified that the *ε2* allele and *ε2*-involved genotypes may be implicated in MI as protective factors; in contrast, the *ε4* allele and *ε4*-involved genotypes (*ε4/ε3* vs. *ε3/ε3*, and *ε4/ε4* vs. *ε3/ε3*) may be implicated in MI as risk factors. Luc et al. conducted their meta-analysis based on a multicenter population-based case–control study [29]. Population-based articles are more creditable than hospital-based articles and are less frequently performed in other meta-analyses. [18, 29, 40].

Wang et al. observed the genotype *ε4/ε4* had no significant association with MI risks [18]. In addition, Kenji et al. and Prabhat et al. both observed the *ε2* allele and *ε2*-involved genotype (*ε2/ε2* and *ε2/ε3*) had no significant association with MI risks [31, 40]. Because we performed TSA, the disagreements may be because the false-negative error was existed in those studies [18, 31, 40]. In addition, Kenji et al. just enrolled Japanese patients [31] and the articles of Prabhat et al. investigated Indian individuals[40]. For these reasons, we performed subgroup analysis stratified by ethnicity, identifying that the association of MI risks with the *APOE ε2* allele and with genotypes (*ε2/ε2, ε2/ε3*) is weaker in Asian than that in other ethnicities. Furthermore, we performed sensitivity analyses and TSA to obtain a reliable conclusion.

Our study has some limitations. First, despite subgroup analyses and regression, the main sources of heterogeneity remain difficult to identify. Second, our study focused on articles based on case–control design, merely providing the associations between *APOE* polymorphism and MI risks, rather than a causal relationship. Third, we did not retrieve other confounding factors, such as the low-density lipoprotein receptor gene, lifestyle, and gene–gene or gene-environment interactions, because the articles included in this meta-analysis did not provide any information about the other confounding factors.

Despite the limitations above, our study has some strengths. First, up-to-date articles were collected extensively, conferring our study more statistical power to draw valid conclusions on the associations between *APOE* polymorphism and MI risks. Second, the result of sensitivity analysis documented that our conclusions are stable and reliable. Third, in contrast to previous meta-analyses on the association between *APOE* gene polymorphism and MI risks, this is the first study to use TSA to further build reliable evidence to draw conclusions.

In conclusion, the *ε2* allele and *ε2*-involved genotypes, as protective factors, have been implicated in MI. However, the *ε4* allele and *ε4*-involved genotypes (*ε4/ε3* vs. *ε3/ε3*, and *ε4/ε4* vs. *ε3/ε3*) may perform as risk factors for MI.

## Supplementary Information

**Additional file 1**. **Figure S1.** Funnel plot of the association between APOE gene polymorphism and myocardial infarction. (A) ε2 allele; (B) ε4 allele; (C) ε2/ε2 genotype; (D) ε2/ε3 genotype; (E) ε2/ε4 genotype; (F) ε3/ε4 genotype; (G)ε4/ε4 genotype.

**Additional file 2**. **Figure S2.** Trial sequential analysis of the association between ApoE gene polymorphism and myocardial infarction. (A) ε2 allele; (B) ε2/ε2 genotype; (C) ε2/ε3 genotype.

**Additional file 3**. **Figure S3.** Trial sequential analysis of the association between ApoE gene polymorphism and myocardial infarction. (A) ε4 allele; (B) ε3/ε4 genotype; (C) ε4/ε4 genotype.

**Additional file 4**. **Figure S4.** Trial sequential analysis of the association between ε2/ε4 genotype and myocardial infarction.

## Data Availability

The datasets used and/or analyzed during the current study are available from the corresponding author on reasonable request.

## Abbreviations

APOE: Apolipoprotein E
MI: Myocardial infarction
LDL-C: Low-density lipid protein cholesterol
TGs: Triglycerides
SNPs: Single nucleotide polymorphisms
HWE: Hardy–Weinberg equilibrium
NOS: Newcastle–Ottawa scale
CI: Confidence interval
TSA: Trial sequential analysis
RIS: Required sample size
