# Supplementary figures and images for: Meta-analysis of the association between Apolipoprotein E polymorphism and risks of myocardial infarction
Source: BMC Cardiovasc Disord. 2022 Mar 24;22:126. doi: 10.1186/s12872-022-02566-0 (PMC8952226; doi:10.1186/s12872-022-02566-0)

A

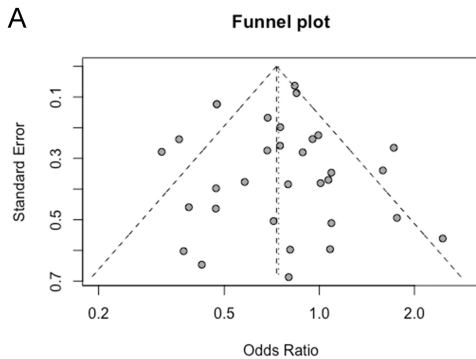

B

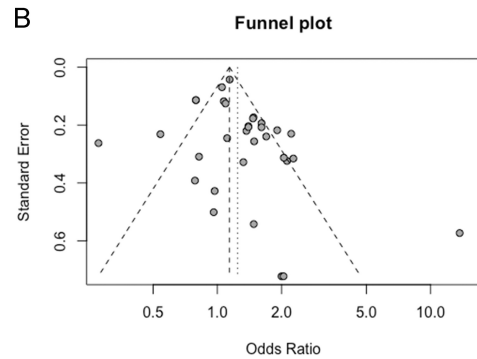

C

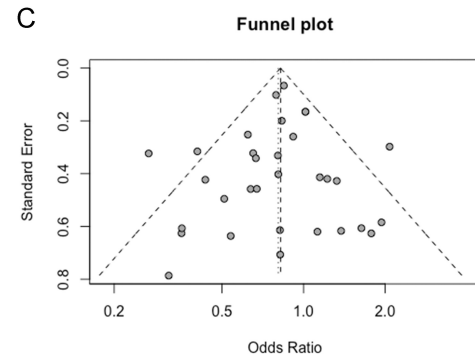

D

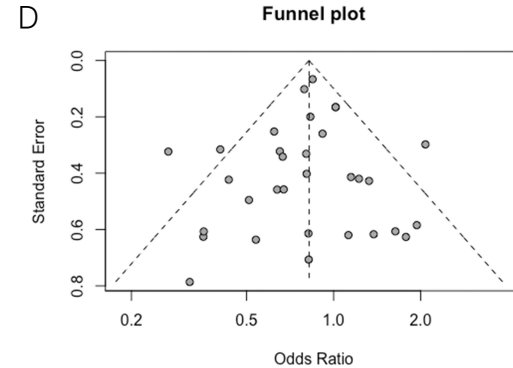

E

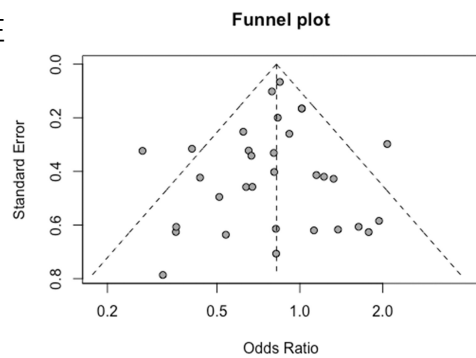

F

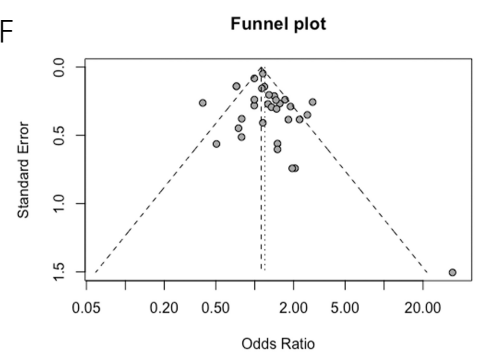

G

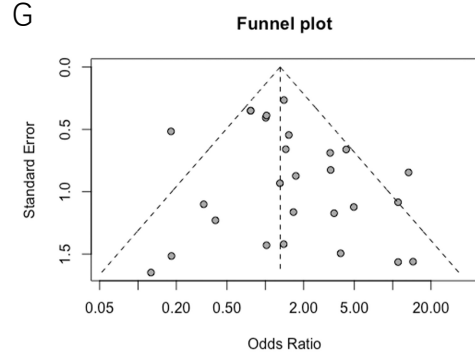

Supplement: Supplementary file 1 — Additional file 1. Figure S1. Funnel plot of the association between APOE gene polymorphism and myocardial infarction. (A) ε2 allele; (B) ε4 allele; (C) ε2/ε2 genotype; (D) ε2/ε3 genotype; (E) ε2/ε4 genotype; (F) ε3/ε4 genotype; (G)ε4/ε4 genotype. [file 12872_2022_2566_MOESM1_ESM.pdf]

A

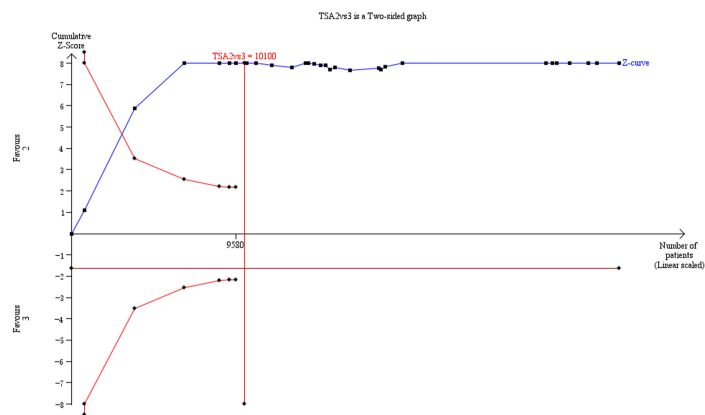

B

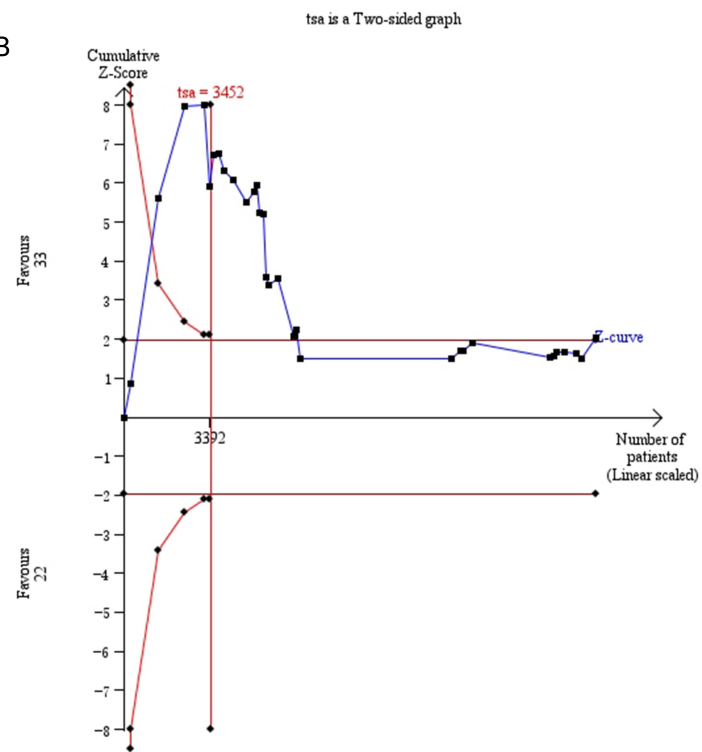

C

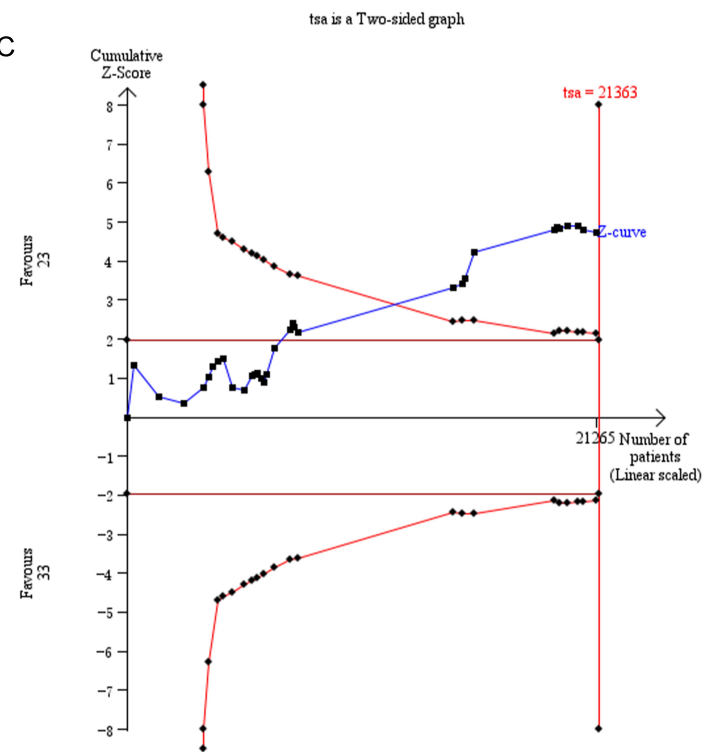

Supplement: Supplementary file 2 — Additional file 2. Figure S2. Trial sequential analysis of the association between ApoE gene polymorphism and myocardial infarction. (A) ε2 allele; (B) ε2/ε2 genotype; (C) ε2/ε3 genotype. [file 12872_2022_2566_MOESM2_ESM.pdf]

A

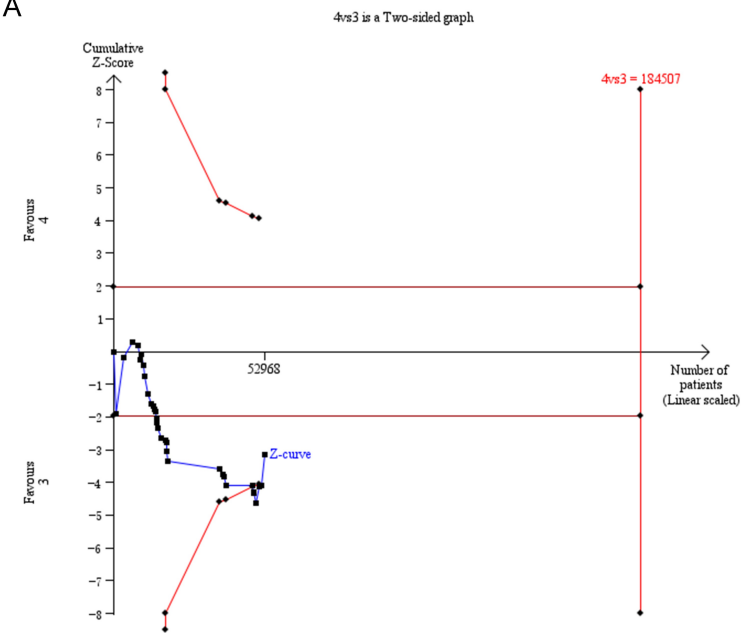

B

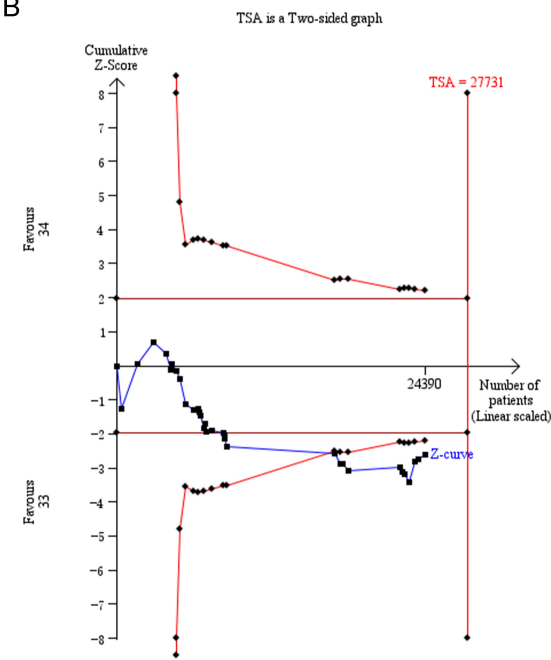

C

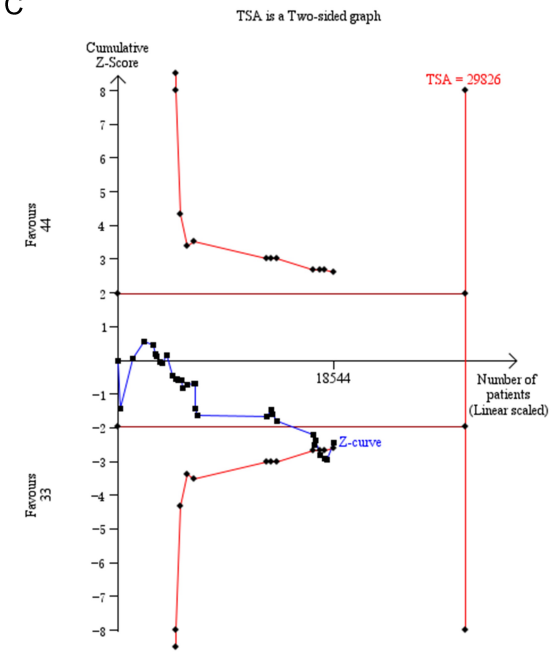

Supplement: Supplementary file 3 — Additional file 3. Figure S3. Trial sequential analysis of the association between ApoE gene polymorphism and myocardial infarction. (A) ε4 allele; (B) ε3/ε4 genotype; (C) ε4/ε4 genotype. [file 12872_2022_2566_MOESM3_ESM.pdf]

TSA is a Two-sided graph

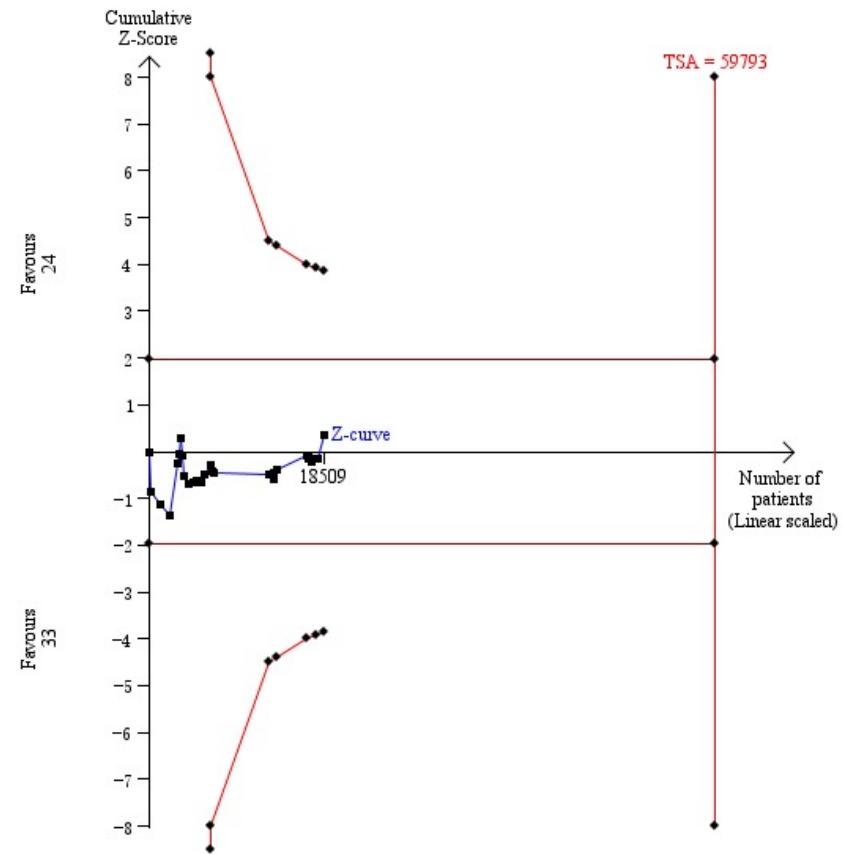

Supplement: Supplementary file 4 — Additional file 4. Figure S4. Trial sequential analysis of the association between ε2/ε4 genotype and myocardial infarction. [file 12872_2022_2566_MOESM4_ESM.pdf]
